# Inhibin Alpha-Subunit (*INHA)* Expression in Adrenocortical Cancer Is Linked to Genetic and Epigenetic *INHA* Promoter Variation

**DOI:** 10.1371/journal.pone.0104944

**Published:** 2014-08-11

**Authors:** Johannes Hofland, Jacobie Steenbergen, Jacoba M. Voorsluijs, Michael M. P. J. Verbiest, Ronald R. de Krijger, Leo J. Hofland, Wouter W. de Herder, Andre G. Uitterlinden, Richard A. Feelders, Frank H. de Jong

**Affiliations:** 1 Department of Internal Medicine, Erasmus MC, Rotterdam, The Netherlands; 2 Department of Pathology, Erasmus MC, Rotterdam, The Netherlands; Virginia Commonwealth University, United States of America

## Abstract

Adrenocortical carcinoma (ACC) is a rare, but highly malignant tumor of unknown origin. Inhibin α-subunit (*Inha*) knockout mice develop ACCs following gonadectomy. In man, *INHA* expression varies widely within ACC tissues and its circulating peptide inhibin pro-αC has been described as a novel tumor marker for ACC. We investigated whether genetic and epigenetic changes of the *INHA* gene in human ACC cause loss or variation of *INHA* expression. To this end, analyses of *INHA* sequence, promoter methylation and mRNA expression were performed in human adrenocortical tissues. Serum inhibin pro-αC levels were also measured in ACC patients. *INHA* genetic analysis in 37 unique ACCs revealed 10 novel, heterozygous rare variants. Of the 3 coding bases affected, one variant was synonymous and two were missense variants: S72F and S184F. The minor allele of rs11893842 at −124 bp was observed at a low frequency (24%) in ACC samples and was associated with decreased *INHA* mRNA levels: 4.7±1.9 arbitrary units for AA, compared to 26±11 for AG/GG genotypes (P = 0.034). The methylation of four proximal *INHA* promoter CpGs was aberrantly increased in five ACCs (47.7±3.9%), compared to normal adrenals (18.4±0.6%, P = 0.0052), whereas the other 14 ACCs studied showed diminished promoter methylation (9.8±1.1%, P = 0.020). CpG methylation was inversely correlated to *INHA* mRNA levels in ACCs (r = −0.701, p = 0.0036), but not associated with serum inhibin pro-αC levels. In conclusion, aberrant methylation and common genetic variation in the *INHA* promoter occur in human ACCs and are associated with decreased *INHA* expression.

## Introduction

Adrenocortical carcinoma (ACC) is a rare malignancy with a poor survival rate [1], [2]. The occurrence of ACC has a female preponderance and a bimodal distribution with an increased incidence in children and in adults over 60 years [3]. Familial ACC occurs in the context of genetic syndromes, such as Beckwith-Wiedemann syndrome [4] and Li-Fraumeni syndrome [5]. Mutations in genes underlying these disorders have also been linked to sporadic ACC formation, especially in the case of *TP53* [6]. The most frequent alteration found in ACC is overexpression of the maternally imprinted IGF-II locus [7]. More recently, mutations in the Wnt/β-catenin pathway have been shown to occur during adrenocortical tumor progression [8]. Genetic causes and the role of chromosomal aberrations in adrenocortical tumorigenesis remain largely unknown.

The inhibin α-subunit (encoded by *INHA*) forms inhibin A or B by coupling to the inhibin βA- or βB-subunits, respectively. Its expression is limited to the gonads, placenta and adrenal cortex. The principal effect of circulating inhibins A and B is inhibition of local activin-induced follicle-stimulating hormone (FSH) secretion in the pituitary gland [9]. In a murine knockout model, the inhibin α-subunit was found to have a tumor suppressive role for gonadal tissue [10] and, after gonadectomy, for the adrenal cortex [11]. Ninety-nine percent of *Inha* −/− mice developed adrenocortical steroid-secreting carcinomas after gonadectomy [11]. Pathways involved in this effect include the differentiation into granulosa cell-like cells with expression of fetal or gonadal markers such as *Gata4*, *Lhr, Fshr* and *Cyp17a1* [12]. *Inha-*related carcinogenesis in mice has also been attributed to decreased activin signalling potential and aberrant expression and effects of TGF-β2 [13], [14].

In man, the function of adrenal inhibins is unknown, although its counterpart activin A has been described to be involved in the zone-specific regulation of adrenocortical steroidogenesis [15]. The evidence for *INHA* as a tumor suppressor in human ACC is conflicting. Several mRNA and protein analysis studies have shown lack of *INHA* expression in a proportion of patients with ACC as well as *INHA* overexpression in another subset [16], [17], [18], [19], [20]. Recently, we reported that serum levels of the free peptide form of the α-subunit, inhibin pro-αC, were increased in patients with adrenocortical carcinomas and that these levels can be utilized as a tumor marker [21]; inhibin pro-αC levels may be useful for the differentiation between malignant and benign adrenocortical tumors as well as for follow-up of individual patients. Although the majority of ACC patients showed increased serum levels of inhibin pro-αC a subset of patients had normal levels, possibly representing the tumors that do not express *INHA* [21].

Several DNA alterations are known to influence gene expression during tumorigenesis. Apart from the genetic changes causing aberrant or absent expression, epigenetic alterations, such as chromatin remodelling and CpG methylation, can affect gene transcription and frequently occur in cancer [22]. Methylation of CpG islands in gene promoter regions can result in transcriptional silencing and loss of gene expression due to interference with the binding of transcription factors [23].

Insights into the regulatory control of *INHA* expression could shed further light on the role of *INHA* in human adrenal tumorigenesis and on the variability of serum free circulating inhibin levels that could function as serum tumor marker in ACC patients. In order to unravel this we investigated the regulatory mechanisms of *INHA* expression in human ACC. Sequencing of the *INHA* gene was undertaken to search for genetic variants that could affect gene function or expression levels. Furthermore, quantitative methylation analysis of the *INHA* promoter was performed in order to study whether methylation of CpGs contributes to the differences in expression. These analyses were coupled to tumor mRNA levels of the inhibin α-subunit and serum concentrations of inhibin pro-αC.

## Materials and Methods

### Sample collection

Paraffin-embedded tissue blocks were collected from the pathological archives of the Erasmus MC. Tissue samples originated from patients operated between 1991 and 2010 in this hospital. The diagnosis of adrenocortical carcinoma was made if the van Slooten index exceeded 8 [24]. Tumor staging was performed according to the European Network for the Study of Adrenal Tumors (ENSAT) staging system [1]. Haematoxylin and eosin-stained slides were evaluated by a pathologist and sections with a high percentage of viable tumor cells were microdissected for further analysis. Tissues excised before 2007 were collected from the pathological archives of the Erasmus MC. Informed consent for the secondary use of surplus tissue was obtained from all patients verbally prior to surgery. Patient refusal to make tissue available for research was recorded in writing and these samples were not included in this study accordingly. From 2007 and onwards, samples were collected in a prospective study of adrenal tumors and informed written consent was obtained from all participants. These samples also included adrenal tissues from patients who underwent adrenalectomy due to renal cell carcinoma, adrenal hyperplasia and adenoma. Tumor sections were collected from viable tumor parts and snap-frozen in liquid nitrogen or dry ice shortly after resection. Pre-operative serum levels of inhibin pro-αC were measured in patient subsets using an enzyme-linked immunometric assay (Diagnostic Systems Laboratory, Webster, TX, USA). The study was performed according to the Dutch regulations on the use of residual tissues and approved by the Medical Ethics Committee of the Erasmus MC.

### DNA sequencing

DNA was isolated with a DNA mini kit (Qiagen, Venlo, The Netherlands) according to manufacturer’s protocol and dissolved in H_2_O. Its concentration was measured using a Nanodrop dispenser (Thermo Fisher Scientific, Waltham, MA, USA).

**Figure 1:**
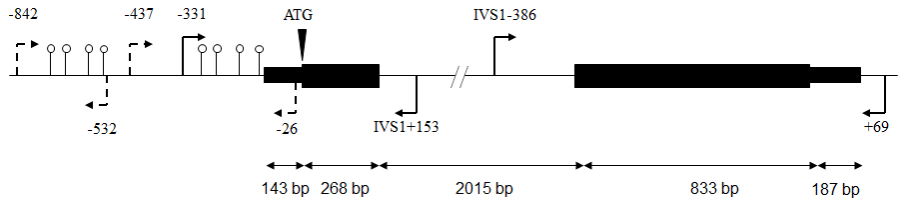
The human inhibin α-subunit (*INHA*) gene. Located at 2q35, *INHA* is composed of two exons separated by a 2 kb intron. The coding sequence is composed of 1101 bps. The regions sequenced in this study are indicated by the continuous arrows. The areas investigated for methylation are depicted by the dashed arrows; CpG dinucleotides successfully characterized are shown as open circles.

The inhibin α-subunit gene, located at 2q35, is composed out of two exons (Figure 1). For DNA analysis of paraffin-embedded tissues primer pairs were selected to amplify regions of 200–250 bp. For freshly frozen tissues regions up to 500 bp could be successfully amplified. Primer locations are summarized in Table 1. The primers covered the coding region, up to −331 bps from the ATG start site (containing the cAMP binding, SF-1 response and GATA elements at −151 to −112 bps [25], [26]) and at least 153 bps of intron adjacent to exon-intron boundaries (Figure 1).

**Table 1:** Primer sequences (5′ to 3′).

sequence	Forward	position
*INHA*	TGTGTGTAGGGAGAAGGTGTT	−331
*INHA*	GGAAGACTGGATGAGAAGGG	−134
*INHA*	TTCTTGCTGCTGACCCC	22
*INHA*	CTGGTGGCCACATCCCTG	IVS1-89
*INHA*	AGAGTGCAGCCCATCATT	IVS1-116
*INHA*	CCATCCATGTAGACACCATTC	IVS1-386
*INHA*	GCACAGCAGCCTCCAATA	422
*INHA*	TCCCCTCTGTACCTGCTCA	600
*INHA*	ATGCCAACTGCCACAGAGTA	776
*INHA*	CGGATGGAGGTTACTCTTTCA	1031
	**Reverse**	
*INHA*	CACCCACCCTCTTCTACC	−99
*INHA*	GCCAGAACAAGTTCCCG	92
*INHA*	TGCTTTTTCTCAAAGTCATCC	IVS1+45
*INHA*	GGGAGACAGAAGCATAAGGA	IVS1+153
*INHA*	GGGGCTCAGAGCTATTGG	451
*INHA*	CGGTGACAGTGCCAGCAG	477
*INHA*	GACATCAGGGGAGTTGAGC	713
*INHA*	AAACTGGGAGGGTACACGAT	857
*INHA*	GAGAAGGTTGGGCACTGTCT	1077
*INHA*	AGATCTGACAGTCCCATGCTC	69 bp 3′ of *INHA*
**methylation**	**Forward**	
*INHA*	AGGAAGAGAGGTTGTTTGGTTTTTTGTTTTTAGGA	−842
*INHA*	AGGAAGAGAGTTGATGTTATTTTTGGATGTGTTTG	−437
	**Reverse**	
*INHA*	CAGTAATACGACTCACTATAGGGAGAAGGCT AACCTTCTAAAACCCCTTTCAATAA	−532
*INHA*	CAGTAATACGACTCACTATAGGGAGAAGGCT TAATAAAAAACTCACACCCTACCCC	−26
**mRNA**	**Forward**	
*INHA*	CCGAGGAAGAGGAGGATGTCT	221
*HPRT1*	TGCTTTCCTTGGTCAGGCAGTAT	293
*GAPDH*	ATGGGGAAGGTGAAGGTCG	1
	**Reverse**	
*INHA*	CGGTGACAGTGCCAGCAG	477
*HPRT1*	TCAAATCCAACAAAGTCTGGCTTATATC	545
*GAPDH*	TAAAAGCAGCCCTGGTGACC	70
	**probe** (FAM-TAMRA labeled)	
*INHA*	TGACTTCAGCCCAGCTGTGGTTCCA	377
*HPRT1*	CAAGCTTGCGACCTTGACCATCTTTGGA	489
*GAPDH*	CGCCCAATACGACCAAATCCGTTGAC	47

PCR amplication was performed in a 30 µl volume of 0.05 U/µl FastTaq polymerase (Roche Applied Science, Almere, The Netherlands), 1 ng/µl DNA, 250 nM forward and reverse primers, 200 µM dNTPs (Amersham Biosciences, Uppsala, Sweden) and buffer containing MgCl_2_ (Roche) in a GeneAmp 9700 (Applied Biosystems, Nieuwerkerk aan den IJssel, The Netherlands) under the following conditions: 7 minutes at 95°C, followed by 40 cycles of 1 minute intervals at 95°C, 56–63°C and 72°C, ending with 10 minutes at 72°C. PCR products were purified by High Pure PCR Product Purification Kit (Roche).

Both forward and reverse PCR primers were used in separate sequence reactions with the BigDye Terminator v1.1 Cycle Sequencing Kit (Applied Biosystems). Three µl of purified PCR product was used with 500 nM of primer in a reaction of 1 minute at 96°C and 25 cycles of 30 seconds at 96°C, 15 seconds at 50°C, and 4 minutes at 60°C. The sequence reaction products were purified with the use of the Dye-Ex 96 Purification Kit (Qiagen) and Micro-Bio-Spin Purification Columns (Bio-Rad, Veenendaal, The Netherlands). Sequence detection was performed using the ABI PRISM 3100 Genetic Analyzer (Applied Biosystems). Sequencher software (Genes Codes Corporation, Ann Arbor, MI, USA) was used for DNA analysis.

### Methylation analysis

One µg of DNA, obtained from frozen samples, was treated with bisulfite using the EZ DNA methylation kit (Zymo research, Irvine, CA, USA), eluted in 100 µl H_2_O and stored at −80°C. Bisulfite-treated DNA in the promoter region of *INHA* was amplified by PCR while a T7 promoter was introduced in the reverse primer. Using EpiDESIGNER software provided by Sequenom (San Diego, CA, USA) two primer sets were designed that covered multiple CpG dinucleotides upstream of the *INHA* start site (Figure 1); the primer sequences are described in Table 1.

The PCR was performed in a 5 µl volume containing 0.04 U/µl HotStar Taq polymerase (Qiagen), 200 µM dNTPs, 200 nM of both primers, 1 µl of bisulfite-treated DNA, HotStar PCR buffer and H_2_O. After 10 minutes at 95°C, 35 cycles were performed of 30 seconds at 95°C, 30 seconds at 53°C and 45 seconds at 72°C. The reaction ended with a 7 minute annealing step at 72°C. After confirmation of PCR product on a 2% agarose-containing gel, the product was treated with 0.3 U Shrimp Alkaline Phosphatase for 20 minutes at 37°C and 5 minutes at 85°C.

Next, *in vitro* transcription and base-specific cleavage was performed in a 7 µl mixture containing 20 U T7 R&DNA polymerase, T-specific cleavage mix, 3.14 mmol/l DTT, RNase A, PCR product and T7 polymerase buffer (Sequenom). The resulting fragments were diluted with H_2_O and 6 mg of CLEAN resin was added for 10 minutes to remove sodium and potassium ions. This mixture was centrifuged at 3000 rpm for 5 minutes and dispensed on a SpectroCHIP with the MassARRAY Nanodispenser RS-1000 instrument (Sequenom). Quantitative methylation was detected by a matrix assisted laser desorption/ionisation time-of-flight (MALDI-TOF) mass spectrometer (MassARRAY Analyzer Compact, Sequenom) and analysis was performed using accompanying EpiTYPER software.

Standard curves for these assays were constructed by assaying mixtures of mixed DNA samples containing 0% to 100% methylation with intervals of 10%. Methylation analysis was successful for 8 CpGs in the *INHA* promoter: located 149, 203, 241, 285, 558, 599, 719 and 751 bps upstream of the *INHA* start site.

### mRNA analysis

Total RNA was isolated from frozen adrenocortical tissues by Trizol reagent (Invitrogen, Carlsbad, CA, USA). Reverse transcriptase and quantitative polymerase chain reaction of *INHA* and housekeeping genes *HPRT1* and *GAPDH* were performed in duplicate as previously described [16]. Sequences of primers and probes have been indicated in Table 1. Expression levels of *INHA* were calculated relative to that of the average threshold cycle (Ct) of *GAPDH* and *HPRT1* using the delta-Ct method and multiplied by 1000.

### Statistical analysis

The dataset for this study is included in Table S1. Analyses of differences between groups were performed with Chi-Square tests, one-way analyses of variance followed by Tukey’s multiple comparison tests or t-tests using Graphpad Prism software (Graphpad Inc, La Jolla, CA, USA). mRNA levels were log-converted before analysis. Correlations were analysed by Spearman’s correlation coefficient. All tests were calculated as two-tailed and a P-level below 0.05 was considered statistically significant.

## Results

### Sequence analysis

The *INHA* sequence was analysed in 37 unique ACC tissues (12 fresh frozen, 25 paraffin-embedded). Results from the sequence analyses have been summarized in Table 2.

**Table 2:** DNA analysis of *INHA* in 37 human adrenocortical carcinomas.

	Clinicalcharacteristics					Sample	Rarevariants‡			Common SNPs							
Patient	Sex	Age†	Cushing	Virilisation	ENSATstage					11893842	35118453	374972575	7588807¶	116399602	12710063	144941390	148455844
1	M	62	–	–	4	frozen				G							
2	F	57	–	–	2	frozen				A/G			T				
3	F	38	–	–	2	frozen							T				
4	M	44	–	–	4	frozen				A/G			T				
5	F	51	–	–	4	frozen				A/G			G/T	G/A	C/T		
6	M	43	–	–	4	frozen								A			
7	F	56	–	–	2	frozen				G							
8	F	54	–	–	2	frozen				G	C/T				T		
9	F	61	–	–	4	frozen	Ser184Phe							G/A		A	
10	M	68	–	–	2	frozen											
11	F	74	+	–	4	frozen				A/G							
12	F	65	–	–	4	frozen											
13	F	33	+	+	4	paraffin	–77G>A	–63A>G	–56G>T	G	T						
14	F	9	+	+	4	paraffin				G							
15	F	69	+	+	4	paraffin								A			T
16	F	40	–	–	2	paraffin	*1G/A										
17	M	54	–	+	2	paraffin						A					
18	F	58	–	–	2	paraffin				G							
19	M	52	+	+	2	paraffin	Ala25Ala								C/T		
20	F	38	+	+	2	paraffin											
21	F	52	+	+	2	paraffin	IVS1-179G>T										
22	M	38	+	+	2	paraffin								G/A			
23	F	33	+	+	4	paraffin											
24	F	35	–	+	2	paraffin	Ser72Phe							A			
25	F	53	–	–	2	paraffin								A			
26	F	56	+	+	2	paraffin											
27	M	42	–	–	4	paraffin					C/T						
28	F	69	–	–	4	paraffin											
29	M	41	+	–	4	paraffin					T				T		
30	F	4	–	+	1	paraffin											
31	F	4	–	+	1	paraffin	IVS1-72C>A										
32	F	39	–	–	4	paraffin											
33	F	27	+	–	1	paraffin											
34	F	76	–	–	2	paraffin				G	C/T			G/A	C/T		
35	F	68	+	+	3	paraffin									C/T		
36	F	64	+	–	2	paraffin											
37	F	68	–	–	2	paraffin	IVS1-9T>C										

† expressed in years.

‡ all detected rare variants were heterozygous.

¶ this SNP was only studied in frozen tissue samples.

In total, sequencing of the *INHA* gene in 37 ACCs revealed 10 novel rare genetic variants in 8 ACCs. One ACC harboured three heterozygous variants (−77G>A, –63A>G and –56G>T) in the 5′UTR, whereas a heterozygous variant directly after the stop codon (*1G>A) was detected in another ACC. We localized three intronic variants, located 179, 72 and 9 bps upstream of the intron 1-exon 2 border. In the coding region of *INHA* we detected 1 synonymous nucleotide change (75C>T, Ala25Ala) and 2 missense variants, in 3 distinct ACCs. The latter both comprised heterozygous C→T variants, at bps 215 and 552, leading to serine to phenylalanine changes at amino acids 72 and 184, respectively.

In our complete series of ACC samples, the –124A>G SNP, rs11893842, was the most prevalent genetic variation with a minor allele frequency (MAF) of 24%, compared to 44% in a reference population (www.1000genomes.org [27]). Furthermore, the minor allele of the intronic SNP IVS1-87G>A (rs116399602) was present in 16% of ACC samples, seemingly higher than in healthy individuals (2.8% MAF). Rs7588807 (IVS1-314G>T) was only measured in the subset of frozen DNA samples. The minor T allele was previously reported to occur in 48% in control subjects; the ACCs showed a lower MAF of 29%. Rs35118453 (−16C>T) and rs12720063 (532C>T) were present at low frequencies, comparable to reference [27]: 9% and 12%, respectively. Rs144941390, rs374972575 and rs148455844 within the *INHA* gene were each detected in one ACC sample.

### Methylation analysis

The first series in which *INHA* promoter methylation was investigated, encompassed DNA from 3 normal adrenal glands and 12 ACCs. For the 4 CpG dinucleotides 558, 599, 719 and 751 bps upstream of the *INHA* start site low methylation ratios were obtained in all samples tested: 4.4±1.1%, 4.5±0.8%, 1.7±0.7% and 2.5±0.6% (mean±SEM), respectively. Furthermore, there were no differences between normal adrenal tissues and ACCs (data not shown). The CpGs in proximity to the start site were methylated to a higher degree in a subset of samples; therefore we analysed an additional 7 ACCs with the downstream primer pair only.

**Figure 2:**
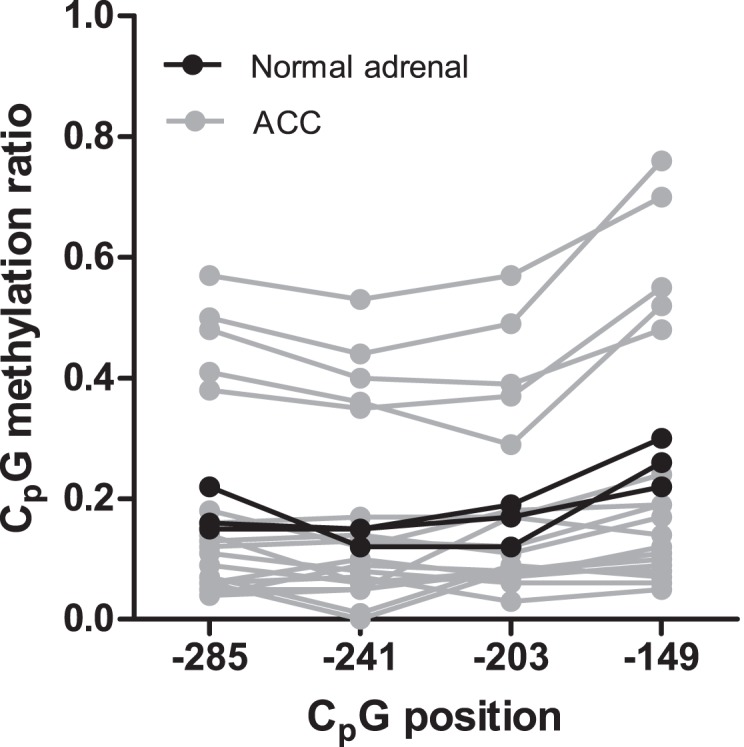
*INHA* methylation analysis in adrenocortical tissues. Quantitative methylation analysis of four CpG dinucleotides in the *INHA* promoter was performed in 3 normal adrenals and 19 ACCs. Individual CpGs are indicated on the x-axis by the bp number located 5′ from the ATG start site. 0 indicates no methylation of DNA whereas 1 indicates that all DNA tested in the tissue sample is methylated. Individual data points are composed of a mean of triplicate measurements.

Results of the methylation analysis of CpGs at −285, −241, −203 and −149 are depicted in Figure 2. Five ACCs had aberrantly high methylation rates of all four proximal CpGs investigated in the *INHA* promoter. Average methylation ratio of these ACCs was 47.7±3.9%, compared to 18.4±0.6% for normal adrenals (P = 0.0052) and 9.8±1.1% for the other ACCs (P<0.0001). The difference in methylation between normal adrenals and the other ACCs was also statistically significant (P = 0.020). The percentage of *INHA* promoter methylation in the ACC samples was not associated with tumor characteristics, such as hormonal overproduction, van Slooten index or ENSAT stage (data not shown).

### Expression analysis

**Figure 3:**
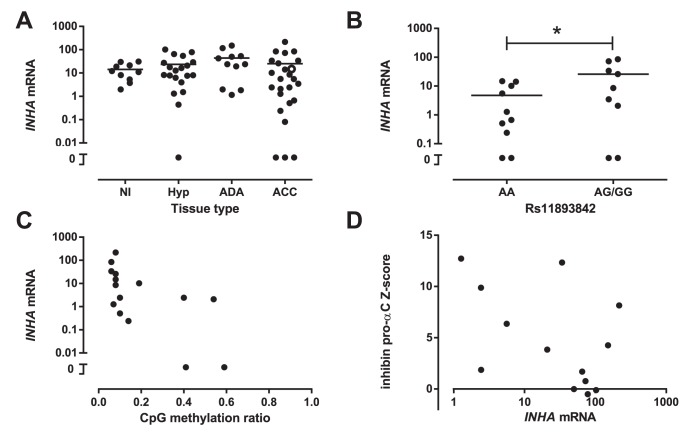
*INHA* mRNA analysis in adrenocortical tissues. (A) Quantitative *INHA* mRNA analysis was comparable in normal adrenals (Nl, n = 10), adrenocortical hyperplasia (Hyp, n = 20), adenomas (ADA, n = 11) and carcinomas (ACC, n = 25). The ACC sample harbouring the S184F variantis displayed as an open circle. Bar represents mean. (B) Variation in rs11893842 (−124A>G) was associated with changes in *INHA* gene expression. Bars represent means, *P<0.05. (C) Negative association between promoter methylation of the *INHA* gene and *INHA* mRNA expression (r = −0.701, P = 0.0036). (D) Lack of correlation between *INHA* mRNA expression and serum inhibin pro-αC Z-scores in ACC patients (r = −0.473, p = 0.10).

*INHA* mRNA expression levels were measured in normal adrenal (n = 10), adrenocortical hyperplasia (n = 20), adenoma (ADA, n = 11) and ACC (n = 25) tissues. The cohort included 10 normal adrenal, 4 adrenal hyperplasia and 14 ACC samples in which *INHA* levels were reported before [16]. The present analysis showed absence of *INHA* expression in 3 ACC whereas the other ACCs demonstrated a wide range of expression from 0.080 to 220 arbitrary units (A.U.). Overall, there were no significant differences between the levels of *INHA* expression in all groups investigated (Figure 3A). Also, mRNA levels of *INHA* in ACCs were not related to tumor characteristics (data not shown).

Of the 3 ACC samples with rare genetic variants in the *INHA* exons, only one was available for expression analysis; this ACC harbouring the S184F change showed a normal level of *INHA* mRNA (15 A.U., Figure 3A, open circle). When stratified for the five common SNPs, only the rs11893842 gene variation was associated with changes in *INHA* mRNA: mean expression in tissues with the AA genotype was 4.7±1.9 A.U., compared to 26±11 A.U. for the AG/GG genotypes (P = 0.034, Figure 3B).

Combined mRNA and methylation analyses were available for 15 tissues. Overall, there was a significant negative association between the average methylation ratio of the proximal CpG islands and *INHA* mRNA expression (Figure 3C, r_s_ = −0.701, p = 0.0036).

Serum inhibin pro-αC levels were available in a subset of patients. There were no significant relations between serum inhibin pro-αC Z-scores on one hand and methylation ratio (n = 9, r_s_ = −0.10, P = 0.81, data not shown) or mRNA expression (n = 13, r_s_ = −0.47, P = 0.10, Figure 3D) on the other. Inhibin pro-αC levels were only available for one ACC patient with rare *INHA* variants: this premenopausal female patient with three rare variants in the 5′UTR of *INHA* had highly increased inhibin pro-αC levels at 3000 ng/l (normal<780 ng/l).

## Discussion

The inhibin α-subunit has been implicated in adrenocortical tumorigenesis since it was observed that gonadectomized *Inha* −/− mice developed adrenocortical carcinomas with almost complete penetrance [11]. Loss of *INHA* expression has been detected in only a small subgroup of human ACCs [16], [17], [18], [20], [28], [29], pleading against a significant tumor suppressor role of *INHA* in human adrenocortical carcinogenesis. This is the first study showing that the large variation in *INHA* expression in ACCs is partly caused by methylation and common genetic variation of the *INHA* promoter and is not due to rare genetic variants.

In the murine knockout model, *Inha* is thought to predispose subcapsular adrenocortical cells to undergo a phenotypic switch to gonadal-like cells [11], [12], [14]. This has been hypothesized to be caused by increased availability of the TGF-β type III receptor betaglycan leading to augmented TGF-β2 signalling [13]. The concomitant rise in circulating gonadotropin levels could ensure proliferation of adrenocortical cells through a cyclin D2-dependent pathway [30]. Although histology of these tumors resembles that of adrenocortical carcinoma in man [11] they are functionally more comparable to estrogen-secreting gonadal-like cells [12]. Whether local knockdown of *INHA* in human adrenocortical cells contributes to adrenocortical tumorigenesis is unknown. Since the occurrence of ACCs shows predominance in postmenopausal women who have increased gonadotropin levels, this mechanism could occur in this subgroup of patients.

Previous expression studies have revealed that the inhibin α-subunit is not expressed in a small subset of ACC samples [16], [17], [18], [20], [28], [29]. In contrast, *in vivo* studies have revealed increased levels of the inhibin α-subunit in serum of patients with ACC [21], [31], [32]. These findings are likely to be a consequence of the wide variation in tumor *INHA* expression levels, in the current study over a 1000-fold. Causes of the loss of or variation in *INHA* expression were unknown.

One previous study has investigated *INHA* genetic variants in ACCs. Longui *et al.* [19] studied pediatric ACC patients with germline *TP53* mutations and found 3 rare, heterozygous *INHA* variants in 6 out of 46 (13%) patients. Of these three novel variants, one (G227A, rs12720061) was subsequently shown to be a common SNP and did not occur in our ACC cohort. Implications of the other two genetic variants (P43A and A257T) are unknown; they were not found in the current investigation of sporadic ACCs. Interestingly, the above mentioned study found loss of heterozygosity (LOH) in the vicinity of the *INHA* gene in eight out of nine ACCs studied [19], suggesting that LOH could cause decreased expression levels. Furthermore, comparative genomic hybridization analyses of human ACCs described sporadic chromosomal loss of the *INHA* region at 2q33-36, with a predominance in childhood tumors [33], [34], [35]. On the other hand, the inhibin α-subunit was previously shown to be overexpressed in pediatric ACCs [21].

In the current study, two novel heterozygous missense genetic variants were detected. The serine to phenylalanine substitutions at amino acids 72 and 184 might affect the activity of the resulting inhibin α-subunit, but since the function of inhibin in the human adrenal gland is unknown [36], it is difficult to investigate the consequences of potentially altered activity. The tumor harbouring the S184F change expressed a normal level of *INHA* mRNA, but function or protein degradation could still be affected by the introduction of the benzyl side ring. The genetic variants located 179, 72 and 9 bps upstream of the intron-exon border might lead to alternative splicing of the *INHA* gene. Importantly, no homozygous variants were detected, pleading against total knockout of *INHA* leading to ACC formation. Unfortunately, we had only one patient with *INHA* variants and concomitantly available serum inhibin pro-αC levels. Here, high serum levels were found despite three variants in the promoter region raising the probability of increased promoter activity. Given the low frequency of *INHA* genetic variants in sporadic and familial ACCs [19], these DNA changes might only be involved in adrenocortical tumorigenesis in a small subset of ACC patients. Several common *INHA* SNPs were detected in our patients. Minor alleles of rs11893842 and rs758807 were found to occur in ACC patients at lower frequencies than previously reported in healthy cohorts, which might suggest that the major alleles of these SNPs play a role in adrenal oncogenesis. Intriguingly, the minor allele of rs11893842, located in the promoter region in close proximity to crucial regulatory sequences [25], [26], was associated with lower levels of *INHA* mRNA expression. The lower frequency of this SNP in ACC samples appears to contradict the hypothesis that *INHA* is a tumor suppressor in human ACC.

Methylation of promoter regions of tumor suppressor genes is a common mechanism involved in carcinogenesis [23]. Decreased *INHA* expression in ACCs could be caused by increased methylation of the *INHA* promoter. Methylation of follistatin, involved in the activin and inhibin signalling pathway as an activin antagonist, was previously found in the human ACC cell line H295R [37]. We now show that a subset of human ACCs (21%) has an increased methylation ratio of several CpGs in the *INHA* promoter, in contrast to the majority of ACCs that show a decreased methylation of the *INHA* promoter compared to normal adrenal tissue. This wide range of methylation presumably accounts for part of the wide *INHA* expression range, as shown by the inverse relationship between *INHA* promoter methylation and *INHA* mRNA expression. This epigenetic modification in the *INHA* promoter and rs11893842 might also be involved in regulatory control of *INHA* expression in the other *INHA*-expressing tissues, i.e. gonads and placenta.

The levels of methylation of the *INHA* promoter and *INHA* mRNA expression were not associated with serum inhibin pro-αC levels. Furthermore, we previously found no association between pro-αC levels and tumor stage or size [21]. Therefore, these levels could be primarily dependent on (post-) translational modifications, tumor cell activity, or clearance from the circulation.

Study limitations include the small sample size, uncertainty about the presence of LOH and whether the novel variants are germ line or somatic. Given the very low incidence of *INHA* genetic variants in this substantial set of 37 ACCs, it is very unlikely that *INHA* mutations play a significant role in ACC formation in man. Although LOH was not studied directly in these samples, the occurrence of heterozygous variants in the ACCs pleads against complete knockout of the gene. Importantly, only homozygous *Inha* knock-out mice developed adrenocortical tumors [11] pleading against tumorigenic potential of single copy number deletion. Finally, paired sequencing of normal and tumor tissue might reveal whether the previously undescribed variants have a germ line or somatic origin, but this was not available for the current study. These findings reveal a novel link between methylation and expression of inhibin. Knockdown of the inhibin α-subunit during ACC formation through methylation might predispose to unopposed paracrine TGF-β or activin action on adrenocortical proliferation or steroidogenesis. Restoring inhibin and also follistatin [37] levels in ACC by demethylating agents might contribute to the antiproliferative and steroidogenic effects seen with DNA methylation inhibitor 5-aza-2′-deoxycytidine in adrenocortical cells [38]. Conversely, methylation of the inhibin promoter might also be a common regulatory mechanism of inhibin expression in gonadal, adrenal and placental tissue and treatment with demethylating agents could be speculated to affect inhibin production in these organs.

In conclusion, aberrant methylation and common genetic variation within the promoter region of the *INHA* gene are associated with *INHA* mRNA expression in human ACC. These genetic and epigenetic *INHA* changes could contribute to human adrenocortical tumorigenesis, similar to the situation in the murine *Inha* knockout model. Rare *INHA* variants do not appear to be involved in the pathophysiology of sporadic ACC.

## Supporting Information

Table S1 **Study dataset.**
(XLSX)
